# Engineering Core-Shell Structures of Magnetic Ferrite Nanoparticles for High Hyperthermia Performance

**DOI:** 10.3390/nano10050991

**Published:** 2020-05-21

**Authors:** Mohamed S. A. Darwish, Hohyeon Kim, Hwangjae Lee, Chiseon Ryu, Jae Young Lee, Jungwon Yoon

**Affiliations:** 1 School of Integrated Technology, Gwangju Institute of Science and Technology, Gwangju 61005, Korea; msa.darwish@gmail.com (M.S.A.D.); duplisona@gist.ac.kr (H.K.); 2 Petrochemical Department, Egyptian Petroleum Research Institute, 1 Ahmed El-Zomor Street, El Zohour Region, Nasr City, Cairo 11727, Egypt; 3 School of Materials Science and Engineering, Gwangju Institute of Science and Technology, Gwangju 500-712, Korea; hj31p@gist.ac.kr (H.L.); ryuchiseon@gist.ac.kr (C.R.)

**Keywords:** magnetic ferrite nanoparticles, modified co-precipitation, core-shell, hyperthermia

## Abstract

Magnetic ferrite nanoparticles (MFNs) with high heating efficiency are highly desirable for hyperthermia applications. As conventional MFNs usually show low heating efficiency with a lower specific loss power (*SLP*), extensive efforts to enhance the *SLP* of MFNs have been made by varying the particle compositions, sizes, and structures. In this study, we attempted to increase the *SLP* values by creating core-shell structures of MFNs. Accordingly, first we synthesized three different types of core ferrite nanoparticle of magnetite (mag), cobalt ferrite (cf) and zinc cobalt ferrite (zcf). Secondly, we synthesized eight bi-magnetic core-shell structured MFNs; Fe_3_O_4_@CoFe_2_O_4_ (mag@cf_1_, mag@cf_2_), CoFe_2_O_4_@Fe_3_O_4_ (cf@mag_1_, cf@mag_2_), Fe_3_O_4_@ZnCoFe_2_O_4_ (mag@zcf_1_, mag@zcf_2_), and ZnCoFe_2_O_4_@Fe_3_O_4_ (zcf@mag_1_, zcf@mag_2_), using a modified controlled co-precipitation process. *SLP* values of the prepared core-shell MFNs were investigated with respect to their compositions and core/shell dimensions while varying the applied magnetic field strength. Hyperthermia properties of the prepared core-shell MFNs were further compared to commercial magnetic nanoparticles under the safe limits of magnetic field parameters (<5 × 10^9^ A/(m·s)). As a result, the highest *SLP* value (379.2 W/g_metal_) was obtained for mag@zcf_1_, with a magnetic field strength of 50 kA/m and frequency of 97 kHz. On the other hand, the lowest *SLP* value (1.7 W/g_metal_) was obtained for cf@mag_1_, with a magnetic field strength of 40 kA/m and frequency of 97 kHz. We also found that magnetic properties and thickness of the shell play critical roles in heating efficiency and hyperthermia performance. In conclusion, we successfully enhanced the *SLP* of MFNs by engineering their compositions and dimensions.

## 1. Introduction

Magnetic ferrite nanoparticles (MFNs) can act as nano-heaters by generating heat under an alternating magnetic field (AMF) and have shown great potential for applications in biomedical fields, such as hyperthermia cancer treatment [1]. For instance, remote heating of tumors by applying AMF has been demonstrated to induce thermal cancer cell death and recognized as a promising tool for cancer treatment. For effective hyperthermia therapy, the temperature of cancerous tissue needs to reach 42–45 °C, while temperatures greater than 50 °C cause damage to cancer cells via thermoablation. Characteristics of MFNs (e.g., size, size distribution and saturation magnetization) and external magnetic field parameters (e.g., duration, frequency and magnetic field strength) are the two main factors affecting the efficacy of the hyperthermia process [2,3]. Hyperthermia behavior is usually quantified in terms of the specific loss power (*SLP*), which refers to amount of the energy converted into heat (*J*) per time (Δ*t*) by a mass of magnetic nanoparticles (*m*) [4,5].

*SLP* = (*C*_p_/*m*) × (d*T*/d*t*) (1)

where d*T*/d*t* is the temperature variation rate. *C*_p_ is the volumetric specific heat capacity, and *m* is the mass of elements per volume.

For magnetic hyperthermia systems, single phase ferrite nanoparticles have been commonly investigated because of their moderate saturation magnetization and ease in synthesis [6]. Such single-phase ferrites are prepared using various methods, such as hydrothermal, thermal decomposition, sol-gel method and co-precipitation processes [6,7,8,9]. Among these, a co-precipitation method is useful for synthesizing single phase MFNs because it is simple, environmentally friendly, and amenable to operation at relatively low temperatures. However, the hyperthermia performance of conventional single-phase ferrites is often insufficient for efficient cancer treatment. Thus, intensive efforts have been made to enhance the heating efficiency of MFNs by controlling their composition, size, and/or structures. This solution-based synthesis method is sensitive to the composition of precursor materials and the reaction temperature, allowing for the synthesis of the bi-magnetic core-shell structured MFNs with controlled size and degree of crystallinity. This method is particularly appealing since it allows the synthesis of heterostructures (i.e., with core and shell composed of different materials) with exquisite control of the sizes and morphologies. This method is based on a two-step synthesis process, where pre-made nanoparticles are used as seeds for the posterior deposition of the shell. MFNs with core-shell structures are expected to have a synergistic effect on hyperthermia performance as bi-magnetic core-shell structures can improve some magnetic properties, such as magnetic saturation (Ms), associated with hyperthermia performance [10]. Specifically, hard and soft phases can exhibit the exchange coupling between them and can increase *SLP* [11]. 

Core-shell magnetic nanoparticles (MNPs) designed to merge the advantageous magnetic properties of soft and hard magnetic materials have shown excellent *SLP* values and tunability of the anisotropy. Anisotropy is the energy required to rotate the magnetization away from the easy direction (atomic orbitals prefer to align at a specific crystallographic direction) [12,13]. The effects of core diameter and shell thickness on the magnetic exchange-enhanced heating efficiency of these core-shell systems have been investigated [12]. As the magnetic phase size decreases to the nano-scale, the magnetic properties of the material greatly decrease owing to the large surface-to-volume ratio, quantum size effects and magnetic interactions [13]. Iron oxide nanocrystals (IONCs) with an average diameter of 19 ± 3 nm had significant specific absorption rate (SAR) values in clinical conditions and reached SAR values up to 2452 W/g_metal_ at 520 kHz and 29 kA/m, which is one of the highest values reported so far for IONCs. In in vitro trials carried out with KB cancer cells, 19 nm IONCs showed efficient hyperthermia performance with cell mortality of approximately 50% when an equilibrium temperature reached 43 °C after 1 h of treatment [14]. Recently, it was shown that by modulating the shell composition and thickness, monodisperse Fe_3_O_4_/Co_x_Zn_1–x_Fe_2_O_4_ core/shell nanoparticles could be designed to provide high heating powers for different field amplitudes and dispersion media conditions. The fine control over the IONCs’ effective anisotropy, which can be provided by the interface coupling between core and shell, could lead to SAR values up to ∼2400 W/g_metal_ for water colloids and ∼1000 W/g_metal_ for immobilized particles at 80 mT and 309 kHz [15]. However, such high SAR values were achieved at high field amplitude and frequency values that are unsuitable for clinical applications.

Biocompatibility is essential for biomedical applications of magnetic nanoparticles. Iron oxides not only show interesting size-dependent magnetic properties and can be functionalized with both organic and inorganic compounds, but also they are considered to be biocompatible and non-toxic, which makes them excellent candidates for biomedical applications and in vivo experiments. The biocompatibility of magnetic nanoparticles generally depends on the coating materials [16,17,18,19]. Until now, only two families of iron oxide nanoparticles have been applied in clinical trials, i.e., those coated with polysaccharides and those coated with silica [16]. The biocompatibility of cobalt containing samples has not been completely assessed for in vivo applications and thus in-depth studies are needed for the evaluation of possible interactions between cells and nanoparticles and the possible effects of the dissolution of the nanoparticles on cell viability, such as increases in Co^2+^ concentrations in the organism [19].

In the present study, for the purpose of developing MFNs with high *SLP* values, we employed a modified and controlled two step co-precipitation process for the preparation of bi-magnetic core-shell MFNs. We have synthesized various core-shell MFNs, i.e., exchange-coupled Fe_3_O_4_@CoFe_2_O_4_, CoFe_2_O_4_@Fe_3_O_4_, Fe_3_O_4_@ZnCoFe_2_O_4_ and ZnCoFe_2_O_4_@Fe_3_O_4,_ and examined their characteristics. Specifically, we performed the systematic studies on synthesis and characterization of bi-magnetic core-shell MFNs by varying the ferrite material types, the core/shell locations, and the dimensions using the co-precipitation method for the production of high *SLP*. 

## 2. Experimental Work

### 2.1. Materials

Iron(III) chloride hexahydrate, iron(II) chloride tetrahydrate, cobalt(II) chloride hexahydrate, zinc(II) chloride, and ammonium hydroxide were purchased from Sigma Aldrich (St. Louis, MO, USA). For the comparison of heat-generation performances of the prepared MFNs, we also purchased and tested commercial iron oxide-based MFNs: i) BNF with a size of 100 nm and Fe of concentration 60 mg/mL (magnetite particles with a shell of dextran dispersed in water from micromod Partikeltechnologie GmbH, Rostock, Germany), ii) SHA30 with a size of 30 nm and SHA15 with a size of 15 nm (functionalized amine iron oxide nanoparticles dispersed in PBS with Fe concentrations of 20 mg/mL and 5 mg/mL, respectively from Ocean Nanotech, San Diego, CA, USA), iii) HyperMAG A, HyperMAG B and HyperMAG C with Fe concentration of 10 mg/mL (magnetic iron oxide nanoparticles with sizes of 10.3, 11.7 and 15.2 nm, respectively, dispersed in water from NanoTherics Ltd, Newcastle, UK), and iv) Resovist with a size of 45–65 nm and Fe concentration of 53.1–58.6 mg/mL (superparamagnetic iron oxide nanoparticles coated with carboxydextran from Meito Sangyo Co., Nagoya, Japan).

### 2.2. Synthesis of Bi-Magnetic Core-Shell MFNs

**Scheme 1:**
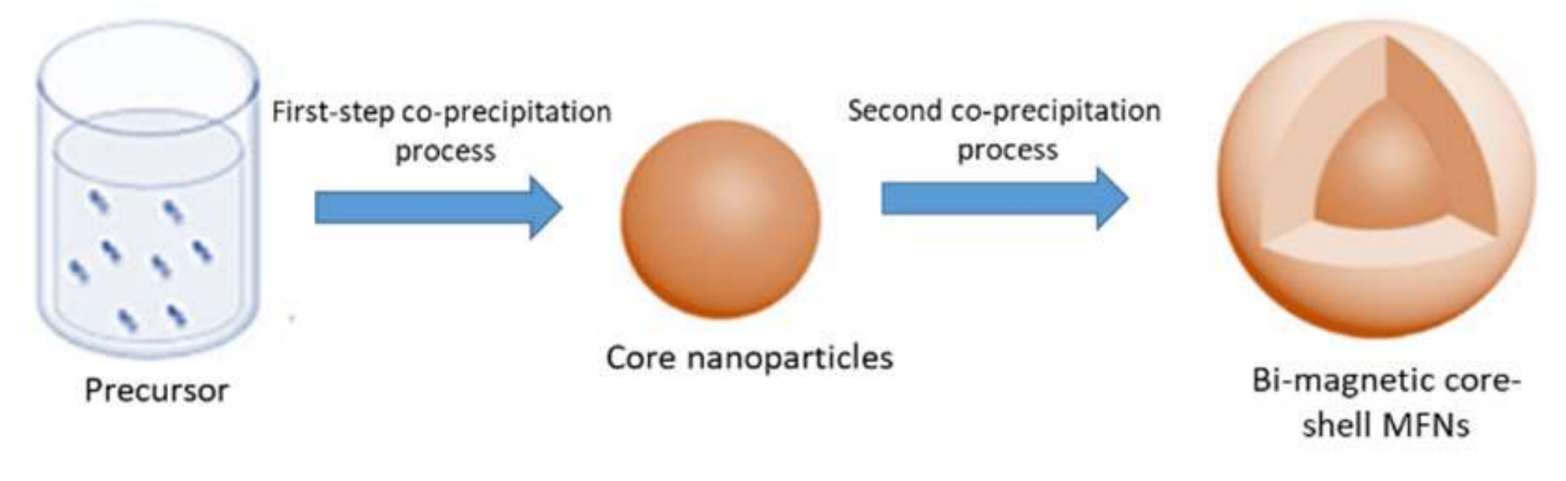
Synthesis of core-shell MFNs.

The synthesis process used herein is a modified version of a previously reported co-precipitation method [6,20]. Core-shell structured MFNs were prepared by the two-step co-precipitation process illustrated in Scheme 1. In the first step, the core nanoparticles based on magnetite (mag), cobalt ferrite (cf) or zinc cobalt ferrite (zcf) were prepared as shown in Table 1.

Precursors were dissolved in 50 mL distilled water and mixed well for 15 min to obtain a homogeneous solution. The temperature was then increased to 60 °C, and maintained for 5 min, to ensure complete homogenous mixing. With vigorous stirring, 20 mL of ammonium hydroxide (30%) was added in a dropwise manner to induce particle growth, followed by additional stirring for 30 min at 60 °C to evaporate any excess ammonia. The black precipitate was washed several times using distilled water to remove possible impurities (e.g., ammonium salts). Magnetic nanoparticles were separated from the medium using a magnetic bar. The products were then dried for 24 h. The second step involved coating the prepared core with a shell layer, and the same procedure described above was employed with the addition of the shell precursors described in Table 1.

### 2.3. Characterization

X-ray diffraction (XRD) of the MFNs was analyzed using an X-ray diffractometer (Rigaku, Tokyo, Japan) equipped with a copper X-ray tube and Cu Kα radiation. Measurements were performed at a scan speed of 4°/min with a 2*θ* ranging from 20° to 70°. 

The crystallite sizes (*D*_p_) of the nanoparticles were calculated using the Scherrer formula [14]:

Crystallite size (*D*_p_) = *Kλ*/(*B*cos*θ*) (2)

where *D*_p_ is the average crystallite size (nm), *B* is the full width at half maximum (FWHM) of XRD peak, *λ* is the X-ray wavelength (1.5406 Å), *K* is the Scherrer constant (shape parameter, 0.89), and *θ* is the XRD peak position.

The lattice parameter, *𝑎*, for all the samples was calculated for the prominent peak using Bragg’s equation:

*𝑎* = 𝑑_hkl_√ℎ^2^+𝑘^2^+𝑙^2^ (3)

where the Miller indices (*h*, *k*, and *l*) represent a series of parallel planes in a crystal with spacing *d*_hkl_

Zeta potential measurements were performed using the zeta-potential and particle size analyzer (ELSZ-2000; Photal Otsuka Electronics, Osaka, Japan). For zeta potential measurement, dried powder (50 mg) of each NP sample was dispersed in 6 mL distilled water to obtain a ferrofluid at a concentration of 8.3 mg/mL at a pH range of 3.5–5. 

The magnetic properties of the samples were measured using a vibrating sample magnetometer (VSM; Lake Shore 7400 series; Lake Shore Cryotronics, Westerville, OH, USA). The metal contents of the nanoparticles were analyzed using inductively coupled plasma-optical emission spectroscopy (ICP-OES; Optima 8300, PerkinElmer, Waltham, MA, USA). The morphology and structure of the samples were characterized using transmission electron microscopy (TEM) and selected area electron diffraction (SAED) (JEM-2100 LaB6, JEOL, Akishima, Tokyo, Japan) at 200 keV. Heating efficiency and *SLP* were measured using the lab-made system. In this setup, a function generator was used to generate a sinusoidal voltage signal which was amplified to the desired power through an AC power amplifier (AE Techron 7224). This amplified signal was applied to a litz wire coil wound around a ferrite core to induce an alternating magnetic field (40~50 kA/m at 97 kHz). High voltage capacitors (CSM 150 capacitor) were used to set the resonant frequency and the temperature of the sample solution was measured using an Osensa optic temperature measurement sensor. The *SLP* value for each sample was calculated using the following equation. Note that we used data obtained from the first few seconds while assuming a quasi-adiabatic regime in this period:

*SLP* = (*C*_p_/*m*) × (*dT*/*dt*) (4)

where d*T*/d*t* is the initial gradient of the time-dependent temperature curve. *C*_p_ is the volumetric specific heat capacity of the sample solution (J/g·°C) (4.184 for water) and *m* is the mass of elements per volume. Dried powder (50 mg) of each NP sample was dissolved in 6 mL of distilled water to obtain a ferrofluid at a concentration of 8.3 mg/mL. In magnetic hyperthermia measurement, the time frame considered was the first 10 s.

### 2.4. In Vitro Cytocompatibility Test

The potential toxicity of the prepared MFNs was examined using an in vitro culture of murine NIH-3T3 fibroblasts and measuring cell viability using the WST assay [21]. The cells were seeded at a density of 5 × 10^4^ cells/well in a 24-well plate and incubated for 1 day in Dulbecco’s modified Eagle’s medium (DMEM) supplemented with 10% fetal bovine serum (FBS) and 1% antibiotics. Then, the culture medium (1 mL) containing the various samples (0.125, 0.25, 0.5, 1, or 2 mg/mL) was added to the cells and further incubated for 1 day. Cells without any treatment of nanoparticles were used as the control. After 1 day of incubation with the particles, the sample solution in each well was removed and the cells were washed with sterile Dulbecco’s phosphate-buffered saline (DPBS). Then, fresh culture medium (0.5 mL) and WST assay solution (0.05 mL) were added to each well and incubated for 2 h. Finally, 0.1 mL of the solution from each well was transferred to a plate and the absorbance (A) at 450 nm was measured using a microplate reader. The cell viability was normalized to the control using the following formula:

*Cell Viability* (%) = (*A_c_* − *A_s_*)/*A_c_* × 100 (5)

where *A_c_* is the absorbance of the control sample and *A*_s_ is the absorbance of sample solution. Experiments were performed in triplicate (n = 3).

Cytocompatibility was performed by staining the cell-containing samples using a Live/Dead staining kit (Invitrogen, USA) according to the manufacturer’s protocol. Fluorescence images of NIH-3T3 fibroblast were randomly taken using a fluorescence microscope (DMI3000B; Leica, Germany). The green-stained cells and red-stained cells represent live and dead cells, respectively. 

## 3. Results and Discussion

### 3.1. Synthesis of Core-Shell MFNs

As the chemical composition and size of magnetic materials play critical roles in magnetization, their fine control is critical for increasing the magnetic hyperthermia heating efficiency of MFNs. In our studies, core-shell structured MFNs, i.e., Fe_3_O_4_@CoFe_2_O_4_ (mag@cf_1_, mag@cf_2_), CoFe_2_O_4_@Fe_3_O_4_ (cf@mag_1_, cf@mag_2_), Fe_3_O_4_@ZnCoFe_2_O_4_ (mag@zcf_1_, mag@zcf_2_), and ZnCoFe_2_O_4_@Fe_3_O_4_ (zcf@mag_1_, zcf@mag_2_), were prepared by a controlled co-precipitation method, which is a simple, environmentally friendly, low-temperature method [6,20]. This method is based on a two-step synthesis process, where pre-made nanoparticles are used as seeds for the posterior deposition of the shell. 

### 3.2. Characterization of the Prepared Core-Shell MFNs

**Figure 1:**
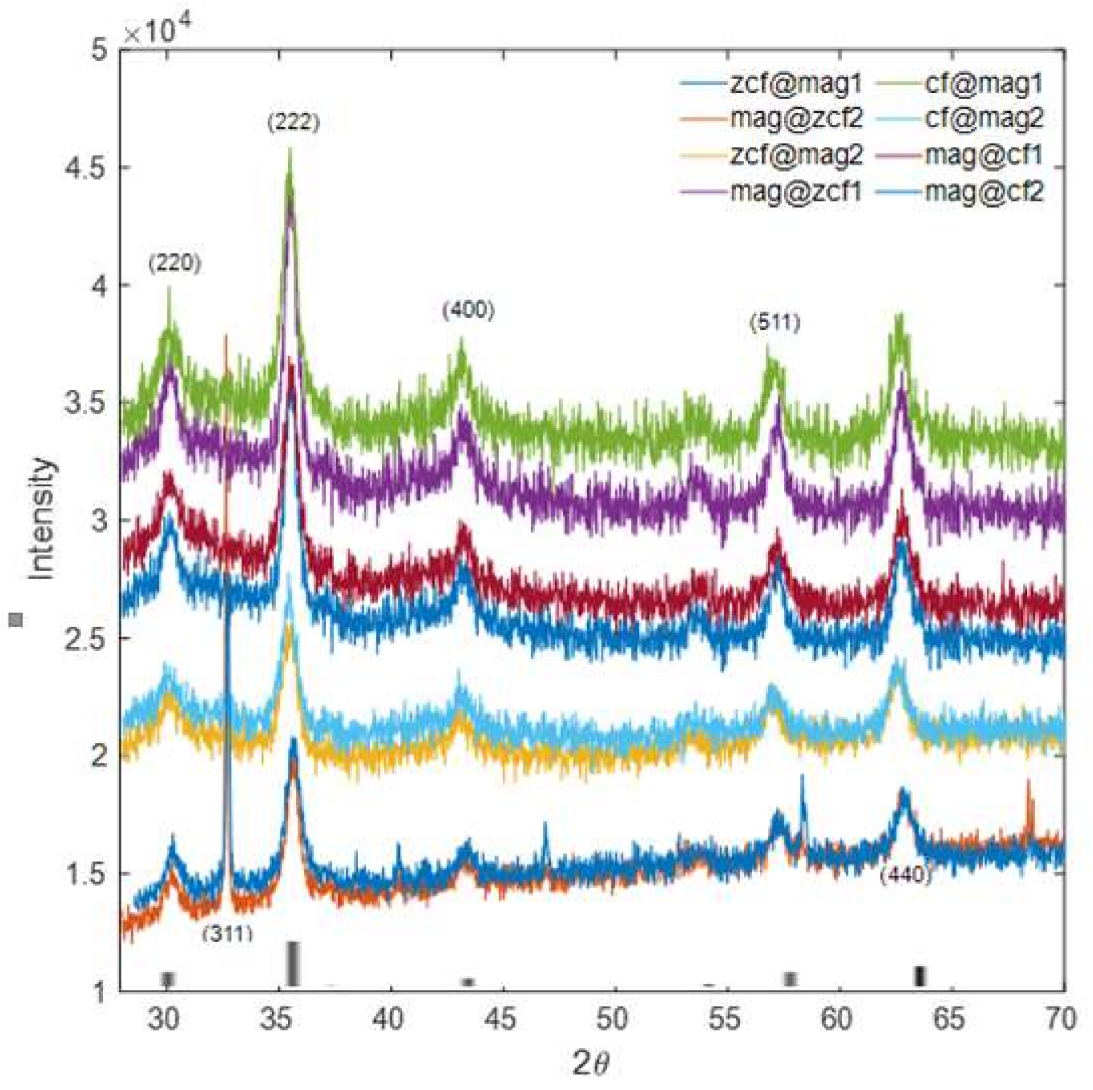
X-ray diffraction (XRD) spectra of various core-shell MFNs.

The crystalline properties of the core-shell MFNs were investigated and compared with the standard (JCPDS data (#221086)) using XRD (Figure 1) [22]. The diffraction peaks indexed to (220), (311), (222), (400), (511), (440) and the spinel ferrite structures were confirmed for the prepared samples. The crystallite sizes (nm) and the associated lattice parameters (Å) for the prepared samples are shown in Table 2. The crystalline properties (e.g., crystallite size and lattice parameter) of the MFNs were calculated based on the broadening of the XRD peak of the maximum intensity. Peak broadening depends on various factors, such as instrumental effects, the strain effect within the crystal lattice, and finite crystallite size. The Bragg peaks are rather broad and weak, likely due to disorder and small crystallite effects. This makes it difficult to make a reliable estimate of the core and shell thicknesses individually. Nevertheless, the crystallite size was found to increase with a decrease in the lattice parameter for MFNs with magnetite shell (mag@cf_2_, mag@cf_1_, mag@zcf_2_ and mag@zcf_1_). This can be explained by the formation of an amorphous shell of iron oxide around the pre-formed core or partial dissolution of the core surface [23].

Crystalline properties (e.g., crystallite size and lattice parameter) of MFNs are generally important for their hyperthermia performance [24]. The magnetization of magnetic nanoparticles decreases with decreasing particle size [25]. As the *SLP* value is dependent on the magnetization and relaxation time of the NP, for a given magnetic material there is an optimum size that will result in enhanced hyperthermia effects [26]. For superparamagnetic nanoparticles, Vreeland et al. observed that the optimum size (around 22 nm) showed maximum *SLP* [26]. The size and shape are also important factors that may affect the heating efficiency. It was revealed that core-shell cube NPs lead to a large heat emission capability, due to their minimized anisotropy with reduced spin disordering [24]. 

Zeta potentials of the prepared core-shell MFNs were also investigated as they are closely associated with the colloidal stability and aggregation of particles (Table 2). The stability of the prepared MFNs is particularly important for their biomedical applications [27]. The highest zeta potential (*ζ*) was obtained from mag@cf_1_ (−29.8 ± 0.9 mV) in a water solution, while the lowest zeta potential was obtained from zcf@mag_2_ (−4 ± 0.5 mV). Higher zeta potentials indicate that the nanoparticle will be stable due to the large electrostatic repulsive forces between the particles. A zeta potential value of approximately ±30 mV is typically considered as the boundary value determining stability in colloidal systems [28].

**Figure 2:**
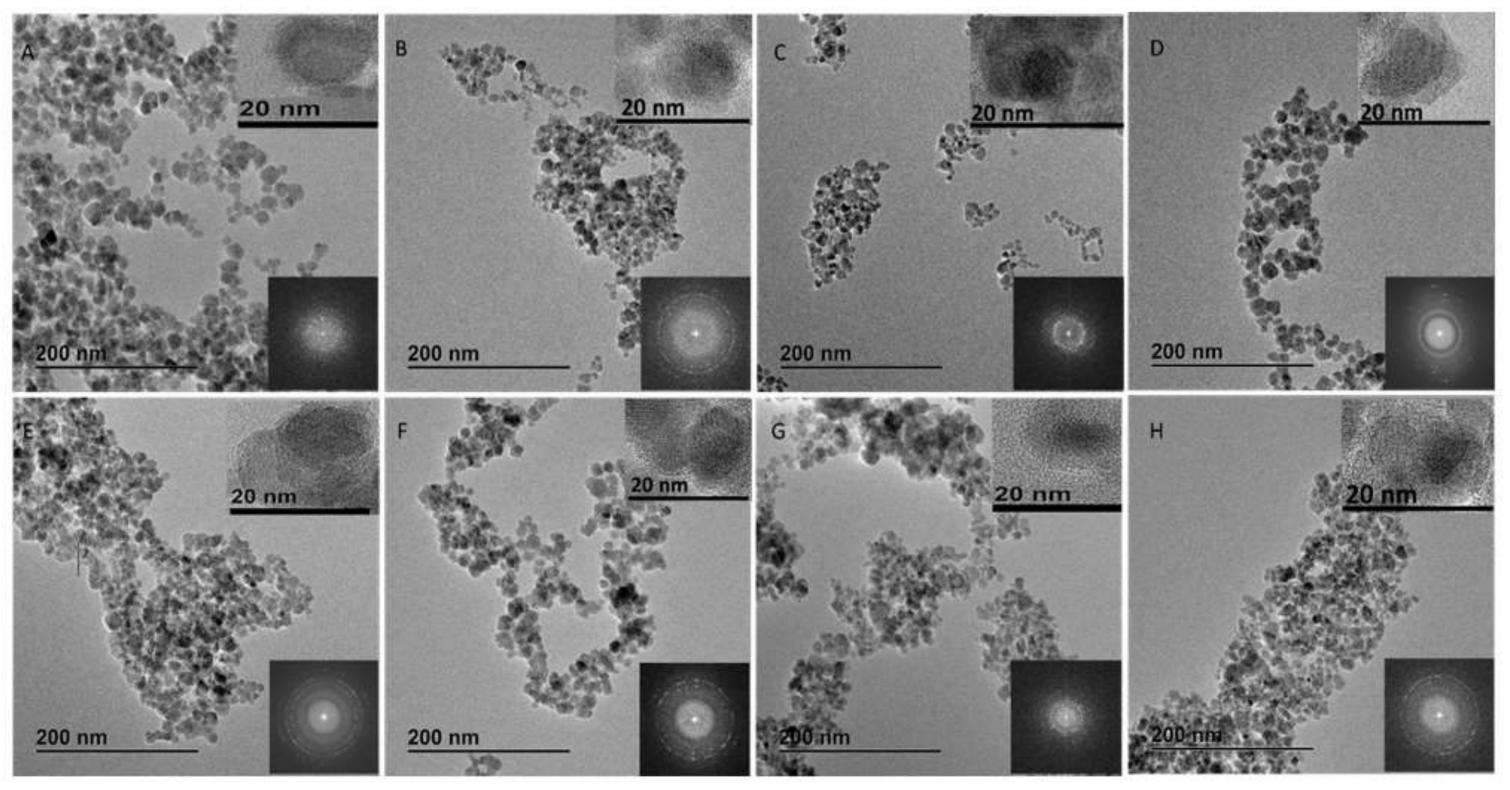
Transmission electron microscopy (TEM) images, size distribution and selected area electron diffraction (SAED) of **A**) mag@zcf_1_, **B**) zcf@mag_2_, **C**) zcf@mag_1_, **D**) mag@zcf_2_, **E**) mag@cf_1_, **F**) mag@cf_2_, **G**) cf@mag_1_ and **H**) cf@mag_2_.

As shown in the TEM images (Figure 2), the core-shell structures of the MFNs prepared by a two-step co-precipitation were confirmed. Magnetic particles were mostly agglomerated, which might be due to high surface energy between the nanoparticles and magnetic dipole–dipole interactions [29,30]. Corresponding selected area electron diffraction (SAED) images of the various nanoparticles displayed the ring characteristics, which indicate a structure composed of small domains with their crystallographic axes randomly oriented with respect to reflections of a spinel phase. The dimensions of the various core-shell MFNs, including average core sizes, and shell sizes, are shown in Table 2. 

The core-shell diameter was determined based on TEM images and the error in determining the average core and shell thicknesses was minimized by analyzing a large number of particles. The size of MFNs can be smaller than the core size, as a result of partial dissolution of the core surface or decrease in aggregation between core nanoparticles by the formation of a thin shell layer [23]. Mag@cf_2_ had the largest size (~16 nm), while zcf@mag_1_ had the smallest (~8 nm). In addition, zcf@mag_1_ had the thinnest core (~7.2 nm) and thinnest shell (~0.7 nm). In contrast, mag@zcf_2_ had the thickest core (~11.9 nm). The shape can significantly alter the magnetic properties. It was observed that the symmetrical particles show high magnetization values, which enhances the heating efficiency of MNPs during hyperthermia heating [10].

**Figure 3:**
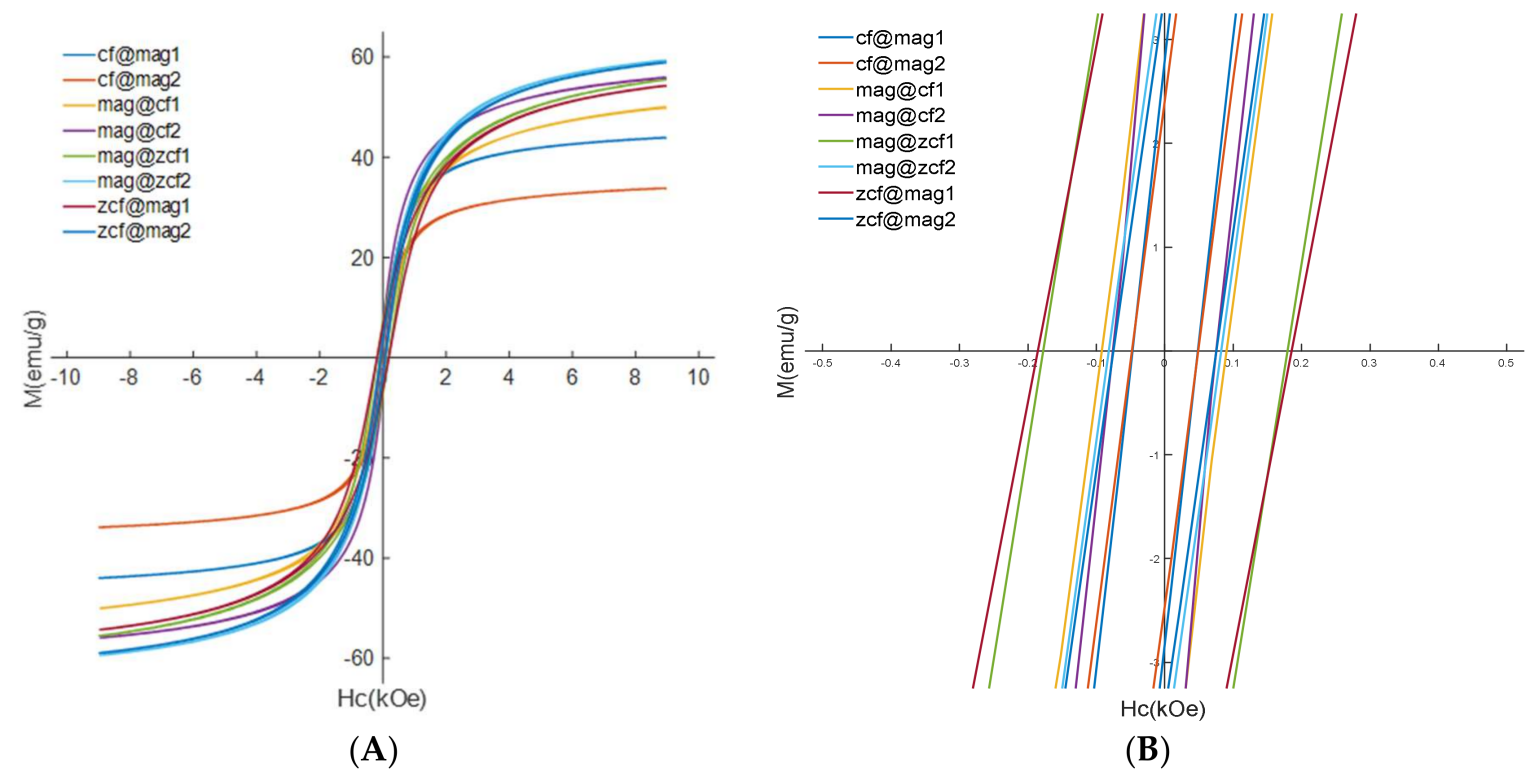
(**A**) Magnetic properties of the prepared core-shell MFNs and (**B**) Closed-up around the zero-field region.

The magnetic properties of the prepared core-shell MFNs were analyzed using VSM at room temperature. As shown in Figure 3, the M–H curves exhibit clear hysteresis loops for all the samples. This observation proves the ferromagnetism of the prepared MFNs. The magnetite nanoparticles show very low coercivity (Hc) value of 40.5 Oe, due to being soft magnetic nanoparticles. The coercivity of core-shell MFNs falls between the values for core nanoparticles and shell nanoparticles. This indicates that the core-shell MFNs have magnetic exchange coupling [31]. The presence of the exchange coupling effect between the hard and soft phases may improve magnetic properties, possibly resulting in the enhancement of the heating efficiency and the *SLP* performance of the core-shell structure [10]. It was found that, for the MFNs consisting of identical core/shell compositions, the Ms decreased with the increase in the core size (Table 2). For example, the Ms for mag@zcf_1_ (core size 7.9 nm) was higher than that for mag@zcf_2_ (core size 11.9 nm). The low magnetization value may be due to either a defect in their crystalline structure with small magnetic domains or their high degree of oxidation and non-magnetic content [32,33,34,35]. Pereira et al. reported the ferromagnetic–superparamagnetic size threshold for cobalt ferrite nanoparticles by tuning the particle size (4.2−4.8 nm) and magnetic properties (Ms 30.6−26 emu/g) [34]. Song et al. simulated coercivity values of conventional core-shell MFN (CoFe_2_O_4_@MnFe_2_O_4_ MFNs) and inverse soft-hard core-shell nanoparticles (MnFe_2_O_4_@CoFe_2_O_4_) with varying core-to-shell volume ratios [35]. They found that the magnetic properties of core-shell nanoparticles could be tuned as a function of the volume fraction of each magnetic phase.

It was observed that Ms (emu/g) was low in the presence of magnetite soft shell in mag@cf_2_, mag@cf_1_, mag@zcf_2_ and mag@zcf_1_ as compared to reversal core-shell MFNs cf@mag_2_, cf@mag_1_, zcf@mag_2_ and zcf@mag_1_, respectively. Saturation magnetization of the prepared core-shell MFNs was improved, except for mag@cf_2_, mag@cf_1_ and mag@cf_2_, as compared to the core nanoparticles (mag, cf or zcf). This may be attributed to the phenomenon that saturation magnetization varies with the shape and size of a particle until it reaches a threshold size beyond which magnetization is constant and is close to the bulk value [34]. 

For our core-shell MFNs, the high Ms was obtained for particles with a thin soft core and a thin hard shell, while the low Ms was obtained for particles with a soft shell and a thick hard core. Khurshid et al. demonstrated that high *SLP* could be obtained by optimization of the shape anisotropy, and not just by saturation magnetization [33]. Reduced remanence, or squareness (SQ), is defined as M_r_/M_s_. When SQ is greater than or equal to 0.5, a material is considered to be composed of a single magnetic domain structure, whereas a material with SQ below 0.5 is considered to have a multi-domain structure. In our study, the SQ values for all the samples were far less than 0.5, indicating the presence of multi-domain structures.

### 3.3. Heating Efficiency of the MFNs

**Figure 4:**
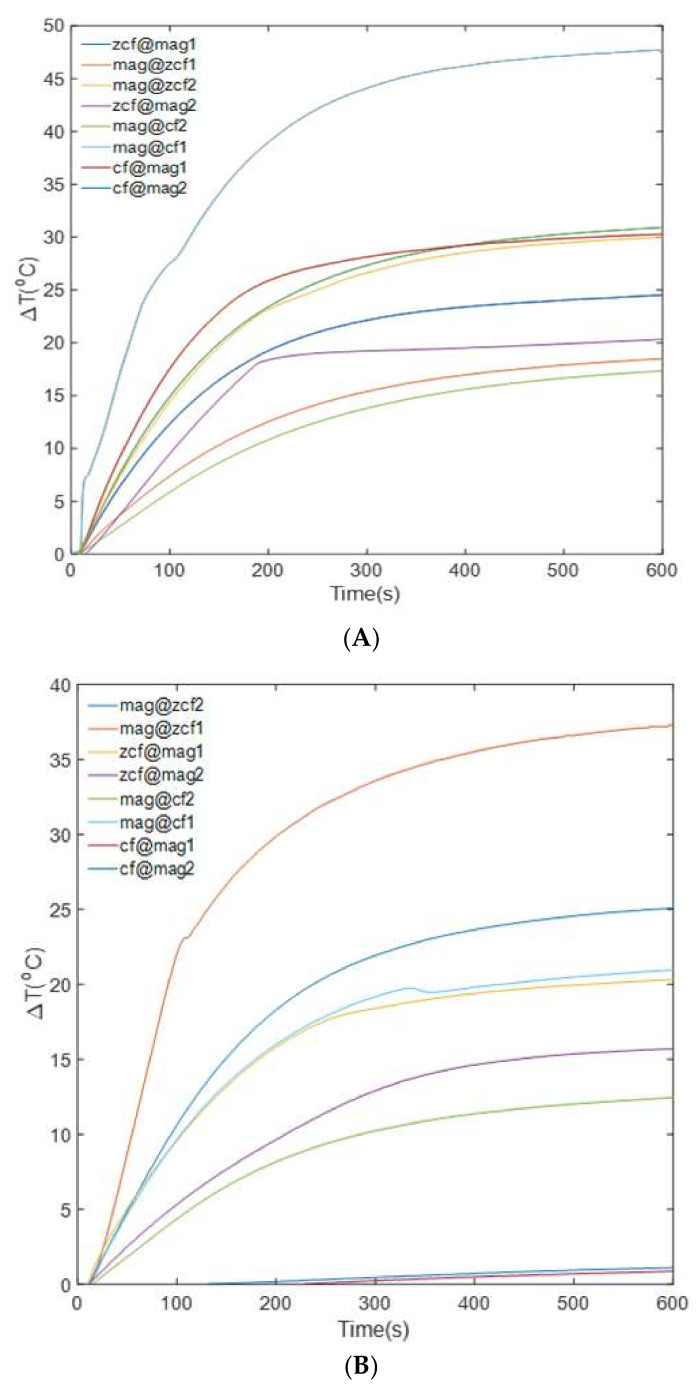
Temperature increases for various core-shell MFNs under magnetic field strengths of (**A**) 50 and (**B**) 40 kA/m.

**Figure 5:**
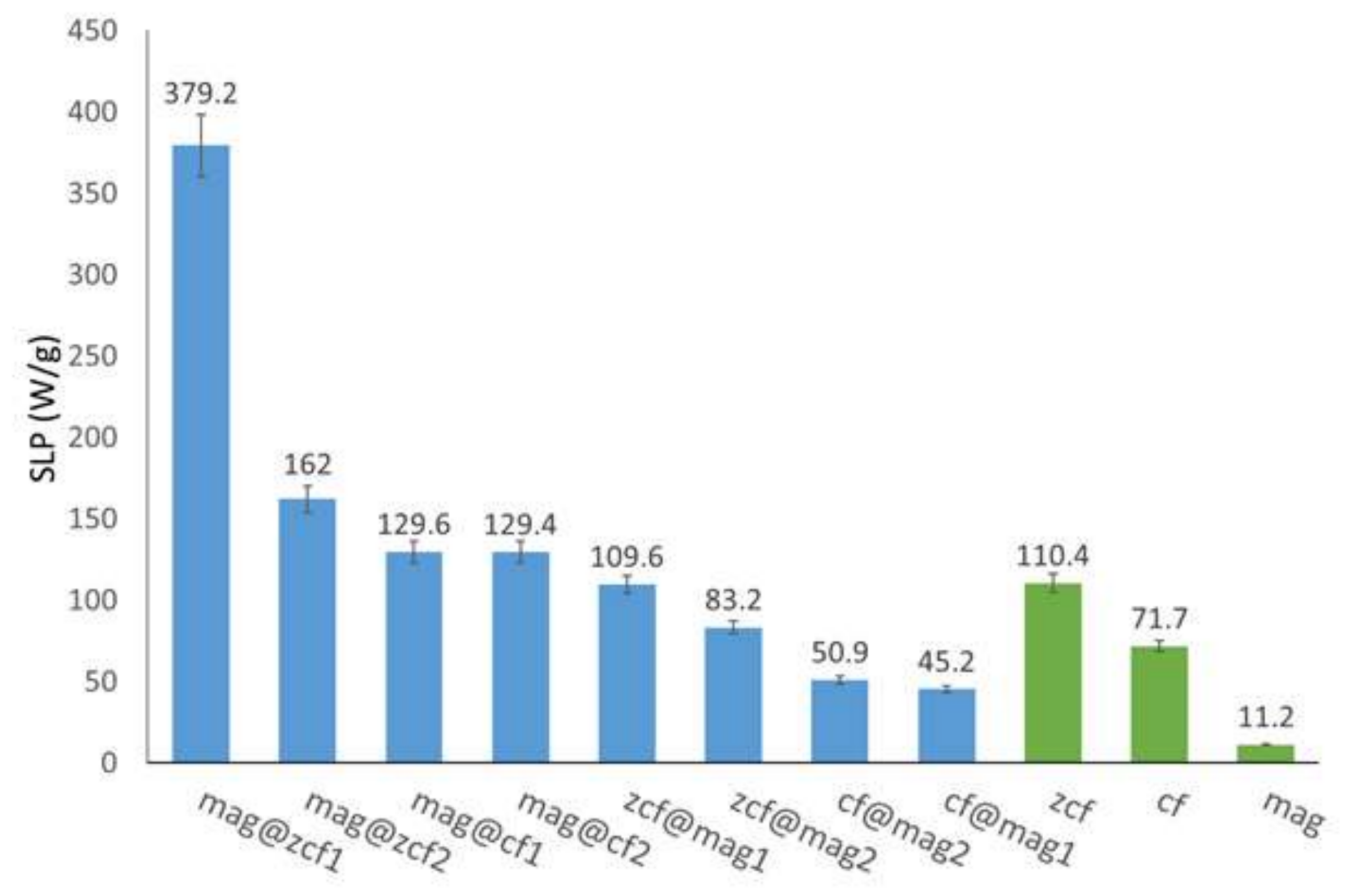
Specific loss powers (*SLPs*) of core nanoparticles and bi-magnetic core-shell MFNs under alternating magnetic field (AMF) with a magnetic field of strength 50 kA/m and frequency 97 kHz.

**Figure 6:**
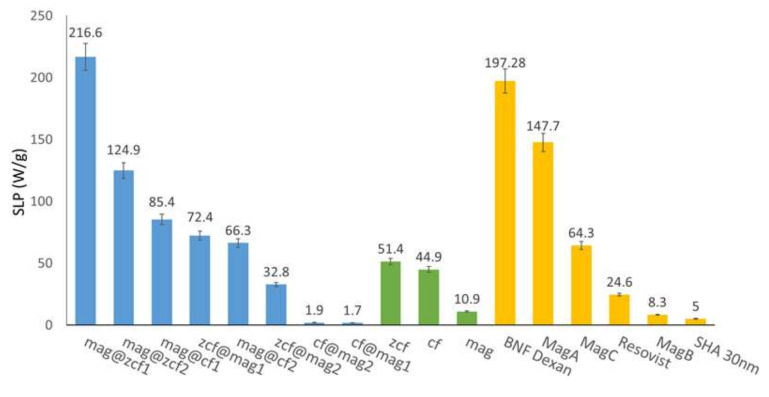
Comparison of *SLP* values of core nanoparticles, bi-magnetic core-shell structured MFNs, and commercial under the action of magnetic field of strength 40 kA/m and frequency 97 kHz.

Importantly, for human exposure, it is pivotal to maintain the product of the magnetic field strength (*H*) and its frequency (*f*) below a threshold safety value known as the Brezovich criterion. A safety limit commonly prescribed is that the product of the frequency and the field amplitude (C = *H* × *f*) should remain below 5 × 10^9^ A/(ms) to minimize any collateral effects of alternating magnetic fields on the human body [22]. The heating efficiency of the prepared core-shell MFNs was investigated by applying AC magnetic fields of various strengths (40 or 50 kA/m) at a frequency of 97 kHz. The values of C in our experiments were calculated to be 3.8 × 10^9^ and 4.8 × 10^9^ A/(m·s) for 40 and 50 kA/m, respectively, which did not exceed the safety limit. Figure 4A,B show the temperature rise (∆*T*) in terms of time (heating curve) for all core-shell MFN samples over a time interval of 600 s. The heating rate and the maximum temperature increased with the increase in the magnetic field strength, as shown by the temperature curves in Figure 4A,B. Under the high magnetic field strength 50 kA/m condition, it was obvious that the highest heating efficiency (47.6 °C) was obtained for mag@zcf_1_, while the lowest heating efficiency (17.3 °C) was obtained for cf@mag_2_ (Figure 4A). This may also be reflected in *SLP* values shown in Figure 5. During the application of the AC magnetic field, the temperature increases were approximately linear at the initial time points (up to 100 s) and gradually slowed down until saturation. Under the condition of low magnetic field strength 40 kA m^−1^, it was also obvious that the highest heating efficiency (36.9 °C) was obtained for mag@zcf_1_, while the lowest heating efficiency (0.85 °C) was obtained for cf@mag_1_ (Figure 4B), which is further confirmed by the *SLP* values (Figure 6). 

Figure 4B shows that cf@mag_1_ and cf@mag_2_ had very low heating efficiency at 40 kA/m. This may be because the field amplitude of 40 kA/m is insufficient to saturate the magnetization of cf@mag_1_ and cf@mag_2_. The soft magnetic phase can be rigidly pinned by the hard magnetic phase at the interface of bi-magnetic core-shell MNPs. Thus, the soft phase becomes difficult to reverse under an external magnetic field, consequently leading to unconventionally enhanced anisotropy. The degree to which this phenomenon plays a role can be readily controlled by tuning the core-shell parameters such as the core-shell ratio, core-shell interface structure, and core-shell composition [13]. The exchange coupling effect between the hard and soft phases in the core-shell structure may improve some magnetic properties, such as coercivity (Hc) and saturation magnetization (Ms), possibly resulting in the enhancement of heat-generation performance of the magnetic NPs [10]. Ideally, the viability of cancerous cells can be reduced to a large extent in a hyperthermia temperature range of 42–46 °C. On the basis of the generated temperature variability, there are two types of hyperthermia therapy: 1) thermal ablation at a temperature >50 °C for a short time period (10–30 min) and 2) mild hyperthermia at a temperature of 42–45 °C (30 min) [36].

*SLP* has widely been used as a parameter to evaluate the amount of absorbed energy per mass of metal nanoparticles under the action of an alternating magnetic field (AMF) [4]. We calculated the *SLP* (W/g_metals_) values based on the total metals content of the MFNs and used the data obtained for the first 10 s while assuming a quasi-adiabatic regime in this period (where there is no heat exchange between the sample and the surroundings). The *SLP* of core-shell MFNs under an external AC magnetic field can be attributed to two power loss mechanisms: Néel relaxation and Brownian relaxation [5]. In the current study, the highest *SLP* value (379.2 W/g_metal_) was obtained from mag@zcf_1_ at 50 kA/m and 97 kHz, where the *SLP* value was proportional to the applied AC magnetic field strength (Figure 5). Improved *SLP* efficiency was observed for the exchange coupled mag@zcf_1_ (379.2 W/g_metal_) compared to the single component magnetite nanoparticles (11.2 W/g_metal_) and the zinc cobalt ferrite nanoparticles (110.4 W/g_metal_). It was reported that an effective exchange coupling occurs when the thickness of the soft magnetic phase falls within a range less than twice the width of the hard-phase domain wall (ca. 10 nm for ferrite MNPs) and when the “softening” or “hardening” effects in a coupled system strongly depend on the ratio of each phase [13]. Overall, heating efficiency with high *SLP* was obtained with core-shell MFNs likely due to hardening effect (the presence of a thin soft magnetite shell compared to a large core size) (mag@zcf_1,_ mag@zcf_2,_ mag@cf_1_ and mag@cf_2_). This result can be attributed to the compositional effects of cobalt, zinc and iron atoms in a large core size, which play a key role in hardening the soft magnetite shell to change anisotropy. Enhancement of heating efficiency was obtained with core-shell MFNs of more elements in the core part, as three elements (Zn–Co–Fe) > two elements (Co–Fe). Lee et al. [37] reported a large increase in *SLP* through tuning of the anisotropy by varying the combination of CoFe_2_O_4_-based core-shell MNPs. Phadatare et al. [38] also reported that CoFe_2_O_4_@Ni_0.5_Zn_0.5_Fe_2_O_4_ core-shell MNPs have high *SLP*. This result was attributed to the compositional effects of nickel and zinc atoms in a shell, which play a key role in softening the hard magnetic CoFe_2_O_4_ core to have optimal anisotropy. The variation in *SLP* values can be attributed to several factors, including sample size, concentrations, and magnitude and frequency of the applied field [2]. Cheon et al. [39] tested conventional and inverse core/shell nanoparticles based on CoFe_2_O_4_ (hard ferrite) and diverse soft ferrites (e.g., Fe_3_O_4_ or MnFe_2_O_4_). They found that the *SLPs* of core/shell nanoparticles were higher than both conventional materials and single-phase nanoparticles of similar sizes. In particular, inverse MnFe_2_O_4_/CoFe_2_O_4_ nanoparticles had outstanding hyperthermia properties. Recently, the fine control of Fe_3_O_4_/Co_x_Zn_1–x_Fe_2_O_4_ core/shell nanoparticles’ effective anisotropy provided by the interface coupling between the core and the shell could lead to *SLP* values up to ∼2400 W/g_metal_ at 80 mT and 309 kHz (safety limit was exceeded so it is not suitable for clinical applications) [15]. The *SLP* values of our core-shell MFNs were compared to those of commercial MFNs under the same conditions (an alternating magnetic field of strength of 40 kA/m and frequency of 97 kHz) (Figure 6). The lowest *SLP* value (1.7 W/g_metal_) was obtained for cf@mag_1_ with a magnetic field strength 40 kA/m and frequency 97 kHz. On the other hand, mag@zcf_1_ had the highest *SLP* value as compared to the other commercial MFNs (BNF, MagA, MagC, Resovist, MagB and SHA30). Improved hyperthermia efficiency was observed for the exchange coupled mag@zcf_1_ (216.6 W/g_metal_) compared to the single component magnetite nanoparticles (10.9 W/g_metal_) and the zinc cobalt ferrite nanoparticles (51.4 W/g_metal_). Due to the high *SLP* under the magnetic field parameters and the achievement of a saturation temperature of 45 °C, these particles can be useful for hyperthermia cancer treatment applications. The time needed for the temperature to reach 45 °C is a function of AC magnetic field amplitude, the dispersing medium, and the size and concentration of the magnetic nanoparticles. Localized magnetic hyperthermia using magnetic nanoparticles (MNPs) under the application of small magnetic fields is promising for treating small or deep-seated tumors. The interface exchange coupling between the magnetic components in bimagnetic systems has enabled remarkably large *SLP*. However, the origin of such large *SLP* is still unclear due to the complex interplay of several factors that modulate the energy dissipation process, including the fine-tuning of the magnetization and anisotropy, the presence of interparticle interactions, and the peculiar role of the internal spin structure arising from the intraparticle interactions. It is difficult to predict a priori the heating efficiency, and more experimental work is highly desirable on this point.

**Figure 7:**
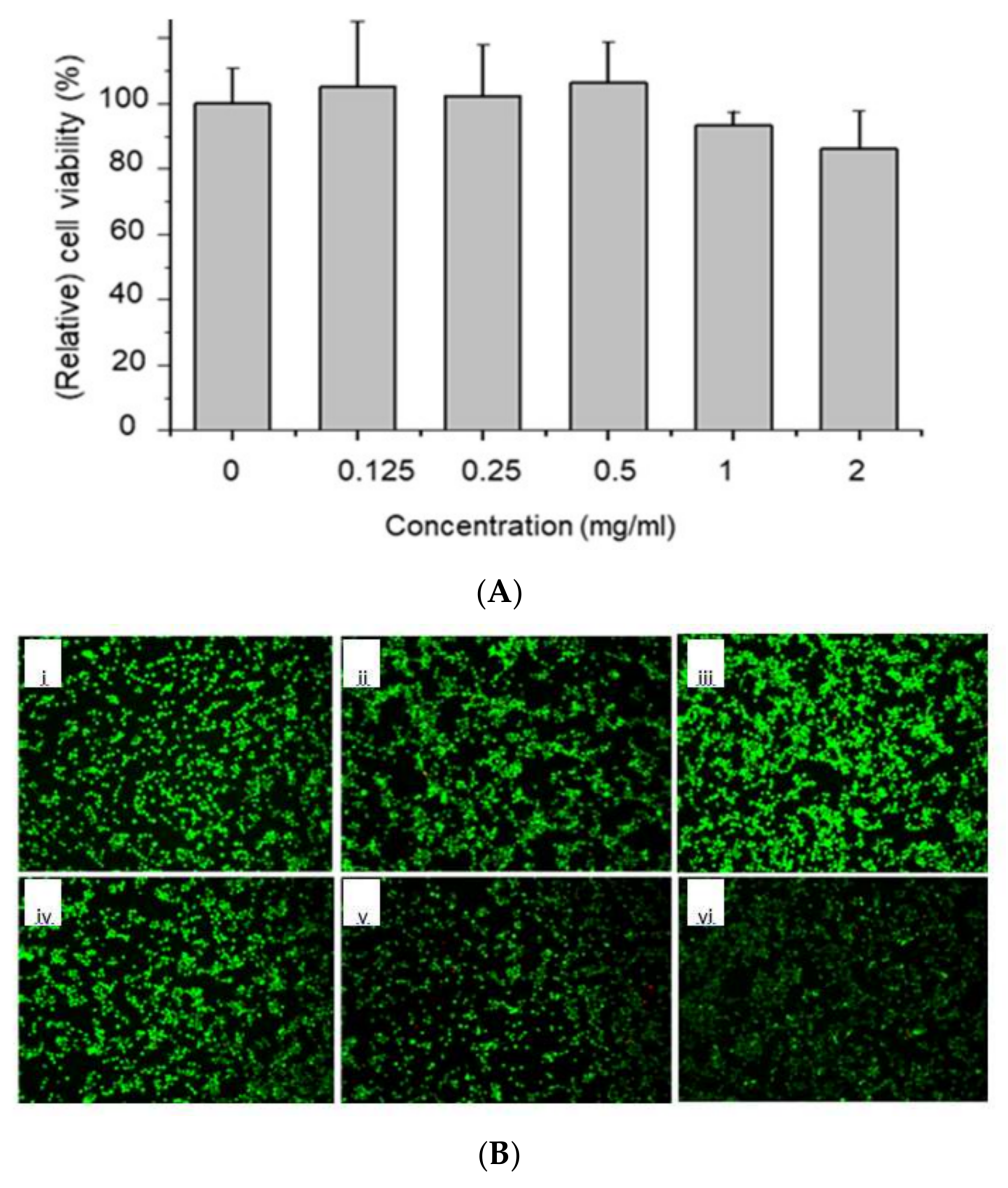
(**A**) Cytocompatibility and (**B**) Live/Dead fluorescence images with i) 0, ii) 0.125, iii) 0.25, iv) 0.5, v) 1 and vi) 2 mg/mL of mag@zcf_1_ in vitro culture with NIH-3T3.

The cytotoxicity of the mag@zcf_1_ nanoparticles, presenting the highest *SLP*, was briefly investigated by in vitro culture with NIH-3T3 fibroblasts (Figure 7). The cell viability remained relatively high up to the particle concentrations of 2 mg/mL compared to the control sample (not treated with any particles). Cytotoxicity is a dose-dependent parameter, and at high particle concentrations (1 and 2 mg/mL) the cell viability slightly decreased. Live/Dead staining of the cells after incubation with various amounts of the mag@zcf_1_ nanoparticles showed good cell viability, as most cells were stained live with minimal red stained cells. Altogether, the results indicate that the mag@zcf_1_ nanoparticles have low cytotoxicity.

## 4. Conclusions

In this study, eight different bi-magnetic ferrite core-shell nanoparticles, including Fe_3_O_4_@CoFe_2_O_4_, CoFe_2_O_4_@Fe_3_O_4_, Fe_3_O_4_@ZnCoFe_2_O_4_ and ZnCoFe_2_O_4_@Fe_3_O_4_, were prepared using a controlled two-step co-precipitation process and characterized to achieve MFNs of high hyperthermia performance. The highest *SLP* of 379.2 W/g_metal_ was obtained for mag@zcf_1_ under the application of an alternating magnetic field of strength: 50 kA/m and frequency: 97 kHz. On the other hand, the lowest *SLP* of 1.7 W/g_metal_ was obtained for cf@mag_1_ under the application of an alternating magnetic field of strength: 40 kA/m and frequency: 97 kHz. The highest *SLP* was obtained by mag@zcf_1_ likely due to the hardening effect. This result was attributed to the compositional effects of cobalt, zinc and iron atoms in a large core size, which plays a key role in hardening the soft magnetite shell. We successfully demonstrated that special core-shell structures can improve the heat generation properties in response to an external alternating magnetic field. Specifically, by tuning the magnetic properties in the prepared core-shell structure, the *SLP* at a particular particle size can be optimized and utilized for various biomedical applications such as hyperthermia cancer treatment.

## Figures and Tables

**Table 1:** Nomenclatures and synthetic compositions of various core nanoparticles and core-shell magnetic ferrite nanoparticles (MFNs).

Name	Core	Shell and Precursors						
		Shell	Fe^+3^ (Mole)	Fe^+2^ (Mole)	DW (mL)	Co^+2^ (Mole)	Amm (mL)	Zn^+2^ (Mole)
**mag**	Fe_3_O_4_	-	0.59	0.399	50	-	20	-
cf	CoFe_2_O_4_	-	0.59	0.399	50	0.199	20	-
zcf	ZnCoFe_2_O_4_	-	0.59	0.399	50	0.199	20	0.199
mag@cf_1_	CoFe_2_O_4_ (0.3 g)	Fe_3_O_4_	0.149	0.098	25	-	5	-
mag@cf_2_	CoFe_2_O_4_ (0.1 g)	Fe_3_O_4_	1.19	0.195	25	-	20	-
cf@mag_1_	Fe_3_O_4_ (0.3 g)	CoFe_2_O_4_	0.074	0.049	50	0.024	5	-
cf@mag_2_	Fe_3_O_4_ (0.1 g)	CoFe_2_O_4_	0.149	0.195	25	0.048	5	-
mag@zcf_1_	ZnCoFe_2_O_4_ (0.3 g)	Fe_3_O_4_	0.149	0.098	25	-	5	-
mag@zcf_2_	ZnCoFe_2_O_4_ (0.1 g)	Fe_3_O_4_	0.599	0.097	50	-	20	-
zcf@mag_1_	Fe_3_O_4_ (0.3 g)	ZnCoFe_2_O_4_	0.149	0.098	25	0.048	5	0.049
zcf@mag_2_	Fe_3_O_4_ (0.1 g)	ZnCoFe_2_O_4_	0.149	0.195	25	0.048	5	0.049

**Table 2:** Crystalline properties, zeta potential, size and magnetic properties of the various core nanoparticles and core-shell MFNs.

	Mag	cf	zcf	mag@cf_1_	mag@cf_2_	cf@mag_1_	cf@mag_2_	mag@zcf_1_	mag@zcf_2_	zcf@mag_1_	zcf@mag_2_
Crystallite size ( nm)	9.8	8.7	9.7	11.13	10.71	9.44	10.16	12.32	12.38	9.44	9.45
Lattice parameter (Å)	-	-	-	8.35	8.36	8.38	8.39	8.39	8.25	8.40	8.36
Core-shell diameter (nm))	10 ± 0.3	8 ± 2	25 ± 5	12 ± 1.7	16 ± 0.46	14 ± 0.5	16 ± 0.25	9 ± 1.8	13 ± 0.9	8 ± 1.3	9 ± 2.5
Core diameter (nm)	-	-	-	7.74 ± 1.1	10.6 ± 0.3	7.56 ± 0.27	10.6 ± 0.16	7.9 ± 1.5	11.9 ± 0.8	7.2 ± 1.1	8.01 ± 2.2
Shell thickness (nm)	-	-	-	4.26 ± 0.6	5.3 ± 0.16	6.44 ± 0.23	5.3 ± 0.08	1.05 ± 0.3	1.07 ± 0.07	0.71±0.2	0.99 ± 0.3
Ms (emu/g)	41.93	50.61	50.71	49.84	43.93	55.45	54.25	55.75	33.77	59.30	58.89
Mr (emu/g)	3.8	10.75	10.71	5	5.5	3.4	2.4	6.8	3.7	6.3	2.7
Hc (Oe)	40.5	159.8	225	90	70	50	50	180	80	190	70
SQ	0.090	0.212	0.211	0.10	0.12	0.06	0.04	0.12	0.010	0.106	0.045
Zeta potential (mV)	−34 ± 0.6	−30 ± 0.5	+14 ± 0.6	−29.8 ± 0.9	−13.1 ± 2.5	−25 ± 0.9	−25.1 ± 0.3	−26.9 ± 0.7	−20.5 ± 1.8	+4.8 ± 0.4	−4 ± 0.5
